# Lenalidomide in the Treatment of Young Patients with Multiple Myeloma: From Induction to Consolidation/Maintenance Therapy

**DOI:** 10.1155/2012/906247

**Published:** 2012-07-11

**Authors:** Barbara Lupo, Antonio Palumbo

**Affiliations:** Myeloma Unit, Division of Hematology, University of Torino, AOU San Giovanni Battista, 10126 Torino, Italy

## Abstract

Multiple myeloma is the second most common hematologic malignancy. It accounts for 20,580 new cancer cases in the USA in 2009, including 11,680 cases in men, 8,900 cases in women, and 10,580 deaths overall. Although the disease remains still incurable, outcomes have improved substantially over recent years thanks to the use of high-dose therapy and the availability of novel agents, such as the immunomodulatory drugs thalidomide and lenalidomide, and the proteasome inhibitor bortezomib. Various trials have shown the advantages linked to the use of novel agents in the transplant and not-transplant settings. In particular, this paper will present an overview of the results achieved with lenalidomide-containing combinations in patients eligible for high-dose therapies, namely, young patients. The advantages obtained should always be outweighed with the toxicity profile associated with the regimen used. Therefore, here, we will also provide a description of the main adverse events associated with lenalidomide and its combination.

## 1. Introduction

For many years, the combination vincristine-doxorubicin-dexamethasone (VAD) was the standard induction treatment for young patients with multiple myeloma (MM) eligible for autologous stem cell transplantation (ASCT). Ten years ago patients candidate for transplant used to receive VAD for 4–6 cycles before undergoing transplantation, leading to a partial response (PR) rate ranging from 52% to 63%, with 3% to 13% of complete response (CR) rate. The availability of new drugs, such as thalidomide, lenalidomide, and bortezomib, has dramatically changed the treatment paradigm of this disease and significantly increased the therapeutic options [1].

Lenalidomide is an immunomodulatory drug with higher potency than its analogue thalidomide and without sedative or neurotoxic adverse effects. Differences between lenalidomide and thalidomide activity have been shown in preclinical studies. In comparison with thalidomide, lenalidomide has more antiproliferative activity against hematopoietic tumors, including myeloma cell lines and patients' cells [2, 3], increased inhibition of tumor necrosis factor secretion from activated monocytes, and increased activation of T cells and natural killer cells [4]. In contrast, thalidomide has more antiangiogenic activity than lenalidomide in human models. Both lenalidomide and thalidomide interfere with key events in the angiogenic process, and activities of these drugs can be differentiated qualitatively depending on what component is studied [5]. Lenalidomide is administered orally, and the most common toxicities related to its therapy are neutropenia, thrombocytopenia, and thrombosis [6].

On the basis of two phase 3 clinical trials, lenalidomide has already shown additive and/or synergistic effects when used in association with dexamethasone [7–9]; therefore, this combination is at present indicated for patients with MM, who have received at least one previous therapy. [10, 11]. New and ongoing trials are assessing the benefit of lenalidomide-combination therapies in early phase of treatment. In particular, the present paper will provide an overview of the main latest combinations including lenalidomide, used in young patients either as induction or maintenance treatment.

## 2. Induction Regimens Including Lenalidomide

### 2.1. Standard Approaches

Lenalidomide has been tested in various clinical trials as induction regimen before ASCT. In one randomized trial, lenalidomide in combination with high-dose dexamethasone (RD) showed to be superior to dexamethasone alone [12]. In that study, patients assigned to RD received lenalidomide at the dose of 25 mg/day for 28 days and dexamethasone at the dose of 40 mg/day on days 1–4, 9–12, 17–20, for three 35-day induction cycles. The same dexamethasone dose was administered in the other treatment arm. Patients assigned to treatment with RD had at least PR of 78%, with very good PR (VGPR) of 63%, while the respective figures for dexamethasone alone were 48% and 16% (*P* < 0.001). The 1-year progression-free survival (PFS) was higher with RD (78% versus 52%; *P* = 0.002), so was the 1-year overall survival (OS) (94% versus 88%, *P* = 0.25). Toxicities were higher with RD and were grade 3-4 neutropenia (21% versus 5%; *P* < 0.001), and thromboembolism despite aspirin prophylaxis (24% versus 5%; *P* < 0.001). Considering the good efficacy of the two-drug regimen, 40 eligible patients crossed over to RD arm.

The efficacy of this combination was further improved by reducing the dose of dexamethasone. A phase 3 trial also demonstrated superior survival and lower toxicity with lenalidomide plus low-dose dexamethasone (Rd) [13]. In that trial, patients who were randomly assigned to be treated with RD received lenalidomide at the dose of 25 mg on days 1–21 plus dexamethasone 40 mg on days 1–4, 9–12, and 17–20 of a 28-day cycle, while subjects in the Rd arm received the same dose of lenalidomide and dexamethasone at 40 mg on days 1, 8, 15, and 22 of a 28-day cycle. At least PR rate was 81% in the RD arm and 70% in the Rd arm (*P* = 0.009), with a CR of 5% and 4%, respectively. OS was lower with RD than Rd (2-year OS was 75% versus 87%, resp.). Toxicities were significantly higher with RD compared to Rd, in particular deep-vein thrombosis (DVT) or pulmonary embolism (26% versus 12%; *P* < 0.001), and infections (16% versus 9%; *P* = 0.04). Considering its good toxicity profile, almost all recent trials have used the lower-dose of dexamethasone (namely, 40 mg once weekly or equivalent) and high-dose dexamethasone is no longer recommended in newly diagnosed MM.

The role of Rd induction was also assessed in an Italian study [14]. Four-hundred two patients with newly diagnosed MM were included in the study. Patients received induction therapy with four 28-day cycles of Rd. Patients were subsequently randomized to receive treatment with melphalan-prednisone-lenalidomide (MPR) or high-dose melphalan (MEL200) followed by transplantation. On an intention to treat basis, best responses after Rd induction were 87% PR rate, 52% VGPR rate, and 13% CR. Induction with Rd was well tolerated: the most frequent grade 3-4 adverse events were neutropenia (8%), anemia (7%), infections (4%), and skin rash (5%). The incidence of thromboembolic events was similar in patients randomized to aspirin (2%) or low-molecular-weight heparin (1%) as thromboprophylaxis (*P* = 0.45). These results further confirm the positive role of Rd as induction regimen.

### 2.2. New Approaches

Based on the promising results obtained with lenalidomide in combination with dexamethasone, new combinations including lenalidomide have been tested to further improve outcomes and to achieve maximal tumour reduction.

 A case-matched study compared clarithromycin-lenalidomide plus low-dose dexamethasone (BiRd) with Rd as initial therapy for newly diagnosed MM patients [15]. Seventy-two patients treated with either BiRd or Rd were included in this retrospective analysis. On intention-to-treat, CR rate was significantly higher with BiRd compared with Rd (46% versus 14%, resp.; *P* < 0.001) and so was also VGPR rate or better (74% versus 33%, resp.; *P* < 0.001). BiRd also led to longer median PFS, 48 months with BiRd compared to 28 months with Rd (*P* = 0.044). The 3-year OS was also improved in patients receiving BiRd (90% versus 73%; *P* = 0.17). Main grade 3-4 toxicities of BiRd were hematologic, in particular thrombocytopenia (24% versus 8%; *P* = 0.012). Higher rates of infections (10% versus 17%; *P* = 0.218) and dermatological toxicity (4% versus 13%; *P* = 0.129) were reported with Rd. These results demonstrate the marked benefits of adding clarithromycin to Rd. Future phase 3 trials are needed to confirm and validate these findings.

Richardson and colleagues evaluated the role of bortezomib-lenalidomide-dexamethasone (VRD) in a phase 1-2 study including 66 patients with MM [16]. Bortezomib was administered at the dose of 1.0 or 1.3 mg/m^2^ on days 1, 4, 8, 11; lenalidomide at 15 to 25 mg on days 1–14; dexamethasone at 40 or 20 mg on days 1, 2, 4, 5, 8, 9, 11, 12. In the phase 2 study, bortezomib was given at 1.3 mg/m^2^, lenalidomide 25 mg, and dexamethasone 20 mg. PR rate was 100% in both the phase 2 population and overall, with 74% and 67% each achieving at least VGPR. Forty-two percent of patients proceeded to transplantation. With a median followup of 21 months, estimated 18-month PFS and OS for the combination treatment with/without transplantation were 75% and 97%, respectively. Most common toxicities included sensory neuropathy (80%) and fatigue (64%), with only 27%/2% and 32%/3% grade 2/3, respectively. Moreover, 32% reported neuropathic pain (11%/3%, grade 2/3). Grade 3-4 hematologic toxicities included lymphopenia (14%), neutropenia (9%), and thrombocytopenia (6%). Thrombosis was rare (6%), and no treatment-related mortality was reported. In the light of these results, VRD proved to be effective and well tolerated in newly diagnosed MM.

 The role of VRD was also confirmed in a French phase 2 trial [17]. Thirty-one patients younger than 65 years with newly diagnosed MM were enrolled and received VRD induction treatment (bortezomib 1.3 mg/m^2^ (days 1, 4, 8, 11), lenalidomide 25 mg (days 1–14), and oral dexamethasone 40 mg (days 1, 8 and 14)). Patients underwent ASCT and subsequently received VRD consolidation and lenalidomide maintenance. All patients could be evaluated for response after induction: at least PR was 97%, with a VGPR rate of 26%. Overall, the most common adverse events were sensory peripheral neuropathy (45%), only grade 1-2 events were detected, and gastrointestinal events (42%). Grade 3-4 hematologic toxicities included neutropenia (26%) and thrombocytopenia (6%). No treatment-related deaths were reported. All patients received aspirin, and no DVT nor pulmonary embolism was detected.

 Lenalidomide associated with adriamycin and dexamethasone (RAD; lenalidomide 25 mg days 1–21; infusional adriamycin 9 mg/m^2^ per day on days 1–4; dexamethasone 40 mg days 1–4 and 17–20) was assessed in another phase 2 study [18]. Seventy-five patients with a median age of 57 (range, 35–66) years have been enrolled. In a preliminary analysis, 17 patients were evaluated for postinduction response. Ten subjects (59%) achieved VGPR or better: 6 patients had VGPR and 2 patients each CR and stringent CR. Fifty-one patients were evaluated for toxicity during RAD induction: 31% experienced a serious adverse event, of which 68% were treatment related. Most frequent events were venous thrombosis (*n* = 4), pyrexia (*n* = 3), and syncope (*n* = 2). Neutropenia, extravasation, pleural effusion, and allergic dermatitis accounted for one serious adverse event each. These preliminary results suggest that RAD is a well-tolerated and effective novel induction upfront approach for MM. Incidence of venous thromboembolism was acceptable, while no neurotoxicity was reported.

A more intense combination including four drugs has been recently tested. Promising results were achieved in a phase 2 multicenter study comparing bortezomib-dexamethasone-cyclophosphamide plus lenalidomide (VDCR) with VDR, bortezomib-cyclophosphamide-dexamethasone (VDC), and VDC modified (VDC-mod) [19]. In all arms, the doses of bortezomib and dexamethasone were as follow: bortezomib 1.3 mg/m^2^ on days 1, 4, 8, 11; dexamethasone 40 mg on days 1, 8, 15. In the VDCR, lenalidomide was given at 15 mg on days 1–14 and cyclophosphamide at 500 mg/m^2^ days 1, 8; in the VDC-mod, arm cyclophosphamide was also given on day 15; in the VDR arm, lenalidomide was administered at 25 mg on days 1–14. The four induction regimens were followed by four 42-day maintenance cycles of bortezomib 1.3 mg/m^2^ on days 1, 8, 15, 22. A total of 41 patients were assigned to VDCR arm, and responses were promising, with at least PR of 88% and 24% of CR, and were comparable with the responses detected in the other arms. The toxicity profile associated with VDCR was slightly higher, with serious adverse events detected in 42% of patients, resulting in treatment discontinuation in 19% of the subjects. In particular, the incidence of grade ≥ 3 peripheral neuropathy was 13%, grade ≥ 3 neutropenia was 42%, and grade ≥ 3 thrombocytopenia was seen in 10% of VDCR patients. These results show that VDCR is an effective treatment option, but it is not significantly superior to other less toxic combinations, such as VRD.

A phase 1-2 study evaluated the combination VRD plus pegylated liposomal doxorubicin (VRDD) [20]. Patients received lenalidomide at 15–25 mg on days 1–14, bortezomib 1.3 mg/m^2^ on days 1, 4, 8, 11, dexamethasone 20/10 mg (cycles 1–4/5–8; days of and after bortezomib), doxorubicin 20 or 30 mg/m^2^ (day 4) at 4 dose levels for up to eight 21-day cycles. Response rates in 57 patients who could be evaluated for response were as follows: 96% at least PR, 58% at least VGPR, and 30% CR/near CR. Patients treated at the maximum tolerated dose, which was determined as lenalidomide 25 mg, bortezomib 1.3 mg/m^2^, dexamethasone 20 mg, and doxorubicin 30 mg/m^2^, and completed at least 4 cycles, showed 100% of at least PR. Overall, toxicities were manageable, with grade 3-4 toxicities including neutropenia (18%), thrombocytopenia (7%), infections (16%), and DVT (2%). Grade 3 peripheral neuropathy was detected in 4% of patients, while no grade 4 peripheral neuropathy nor treatment-related deaths was reported. RVDD showed to be well tolerated and highly active in newly diagnosed MM.

The incidence of both venous and arterial thrombosis in newly diagnosed patients increases when lenalidomide is combined with dexamethasone or chemotherapy: thus, thromboprophylaxis is recommended for the first 6 months of therapy. For patients with standard thromboembolic risk, low-dose aspirin is indicated; for patients with high thromboembolic risk, low-molecular-weight heparin is recommended [21].

## 3. Impact of Lenalidomide on Stem Cell ****Collection

Recent reports have focused on hypothetical negative impact of new drugs on stem cell mobilization. Particularly, hematologic toxicity related to treatment with lenalidomide has raised concerns about lenalidomide use in early phase of treatment and on its possible negative effect on peripheral blood stem cell (PBSC) collection in young patients undergoing ASCT.

In fact, patients mobilized with granulocyte colony-stimulating factor (G-CSF) alone after RD induction have collected a median yield of stem cell ranging from 3.1 to 7.9 × 10^6^ CD34^+^/Kg [22–26].

By contrast, the use of cyclophosphamide in addition to G-CSF may overcome this problem and significantly increases the yield of stem cells collected. Different retrospective published trials on lenalidomide induction have in fact shown a median yield of PBSC ranging from 6.3 to 14.2 × 10^6^ CD34^+^/Kg. These data support the idea that RD is probably not a significant burden for an adequate stem cell collection, especially if the duration of induction with RD is short [22–26].

## 4. Consolidation and Maintenance Approaches ****Including Lenalidomide

Consolidation and maintenance therapies have the potential to improve the results achieved after induction treatment and transplantation [27]. Although preliminary data showed that lenalidomide as consolidation/maintenance therapy after ASCT improved responses, the role of lenalidomide treatment as alternative to ASCT still remains uncertain, and further studies are needed.

Consolidation treatment with MPR has been tested in the Italian study, comparing MPR (six 28-day cycles of melphalan (0.18 mg/Kg days 1–4), prednisone (2 mg/Kg days 1–4), and lenalidomide (10 mg days 1–21)) versus tandem MEL200 (melphalan 200 mg/m^2^) with stem cell support [14]. Response rates were similar: at least VGPR rate was 60% with MPR versus 58% with Mel200, including a CR rate of 20% versus 25%, respectively. After a median followup of 26 months, 2-year PFS was 54% in MPR and 73% in MEL200 (HR = 0.51, *P* < 0.001) and this benefit was maintained in the subgroup of patients with standard- or high-risk cytogenetic features, with a 2-year PFS of 46% in the MPR group versus 78% in the MEL200 group in patients with standard risk (HR = 0.57, *P* = 0.007), and 27% for MPR versus 71% for MEL200 in high-risk patients with *t*(4; 14) or *t*(14; 16) or del17p abnormalities (HR = 0.32, *P* = 0.004). The achievement of CR prolonged PFS, and this was more evident in the Mel200 arm. Two-year PFS in patients who achieved CR was 66% in MPR versus 87% in Mel200 group; 2-year PFS in patients who achieved PR was 56% versus 77%, respectively. PFS was significantly prolonged in patients who received a double ASCT. During consolidation therapy, the incidence of grade 3-4 neutropenia (89% versus 55%), infections (17% versus 0%) and gastrointestinal complications (21% versus 0%), was higher in MEL200 patients (*P* < 0.001). This is the first report showing a PFS advantage for ASCT in comparison with conventional therapies including novel agents; however, longer followup is needed to draw definitive conclusion. To date, more information is available on the use of lenalidomide as maintenance therapy. While maintenance including alkylating agents and interferon showed no significant impact on OS [28, 29], thalidomide maintenance seems to be an effective strategy to improve survival [30–33]. However, prolonged exposure to thalidomide may cause cumulative toxicity. Because of its better safety profile, lenalidomide could be an optimal alternative option to thalidomide as maintenance therapy.

In a phase 3 randomized double-blind, placebo-controlled study (CALGB 100104), patients with non-progressive disease after a first line ASCT were randomized to receive placebo or lenalidomide at starting dose of 10 mg daily, escalated to 15 mg daily after 3 months [34]. Final analysis showed that lenalidomide monotherapy maintenance initiated at 100–110 days after ASCT and continued until disease progression, considerably delays time to progression compared with placebo (median time to progression 42.3 months in the lenalidomide maintenance arm versus 21.8 months in the placebo arm), with a good safety profile and low discontinuation rate due to adverse events (12% in the lenalidomide group versus 2% in the placebo group). Furthermore, significant improvements in time to progression were observed in the group receiving lenalidomide maintenance regardless of *β*2 microglobulin levels or choice of induction therapy (thalidomide or lenalidomide).

In the IFM2005-02 study, MM patients younger than 65 years of age, with at least standard disease after a first line ASCT, were randomly assigned to receive consolidation with lenalidomide (25 mg daily, 21 days/month, for 2 months) followed by maintenance with placebo or lenalidomide (10 to 15 mg daily) until relapse [35]. Results showed that lenalidomide maintenance significantly improved PFS, with a median PFS of 42 months from randomization in the lenalidomide group versus 24 months in the placebo group (*P* < 0.01). This benefit was observed regardless of risk factors, such as cytogenetic profile (del 13, + or −), *β*2 microglobulin levels, or response after transplantation. Preliminary data published indicated that this regimen is well tolerated, with a discontinuation rate for serious adverse events similar to placebo.

Of note, both the CALGB and IFM2005-02 studies detected an improvement in PFS with lenalidomide maintenance, but only the CALGB study reported also an OS benefit with this approach [34, 35].

 Recently, the higher incidence of second cancer in patients treated with lenalidomide for long time led to reconsidering the benefit and the duration of lenalidomide maintenance. In both the CALGB 100104 and IFM2005-02, the rate of second primary malignancy was 8%. An analysis of pooled data from 2459 patients enrolled in different studies including maintenance with lenalidomide was performed. Not all the studies showed an increased second cancer risk, whereas different studies confirmed that the benefits achieved with lenalidomide maintenance outweigh the risk to develop a second primary malignancy [36]. Despite the results presented above, a longer period of followup is required to draw definitive conclusion on the role of lenalidomide as consolidation/maintenance therapy, to better define the impact of lenalidomide maintenance on OS and its cumulative toxicity and to establish the optimal duration of lenalidomide maintenance therapy.

## 5. Conclusions

Considering its dual mechanism of action of lenalidomide, comprising tumoricidal effects which rapidly reduce the MM tumor burden, and its immunomodulatory effects which enhance the immune function and maintain disease suppression, this novel agent showed to be effective and safe in patients with newly diagnosed MM. In particular, the combination Rd proved to be a good induction treatment in this setting of patients.

Based on the promising results achieved with Rd, ongoing trials are now testing this regimen in combination with other agents upfront with the aim to increase the tumoricidal effect. Preliminary results showed the positive role of Rd in association with clarithromycin, bortezomib, adriamycin or cyclophosphamide.

In addition, studies are under way to assess the role of long-term treatment with lenalidomide as consolidation/maintenance treatment after ASCT, but longer followup is necessary to draw any definitive conclusion.
